# Behind the scenes: How RNA orchestrates the epigenetic regulation of gene expression

**DOI:** 10.3389/fcell.2023.1123975

**Published:** 2023-01-25

**Authors:** Arianna Mangiavacchi, Gabriele Morelli, Valerio Orlando

**Affiliations:** BESE Division, King Abdullah University of Science and Technology (KAUST), Thuwal, Saudi Arabia

**Keywords:** RNA, non coding RNA (ncRNA), epigenetics, gene expression, chromatin

## Abstract

Non-coding DNA accounts for approximately 98.5% of the human genome. Once labeled as “junk DNA”, this portion of the genome has undergone a progressive re-evaluation and it is now clear that some of its transcriptional products, belonging to the non-coding RNAs (ncRNAs), are key players in cell regulatory networks. A growing body of evidence demonstrates the crucial impact of regulatory ncRNAs on mammalian gene expression. Here, we focus on the defined relationship between chromatin-interacting RNAs, particularly long non-coding RNA (lncRNA), enhancer RNA (eRNA), non-coding natural antisense transcript (ncNAT), and circular RNA (circRNA) and epigenome, a common ground where both protein and RNA species converge to regulate cellular functions. Through several examples, this review provides an overview of the variety of targets, interactors, and mechanisms involved in the RNA-mediated modulation of loci-specific epigenetic states, a fundamental evolutive strategy to orchestrate mammalian gene expression in a timely and reversible manner. We will discuss how RNA-mediated epigenetic regulation impacts development and tissue homeostasis and how its alteration contributes to the onset and progression of many different human diseases, particularly cancer.

## 1 Introduction

The term *epigenetics*, initially coined by Conrad Waddington from the ancient Greek prefix *ἐπι-* (epi-, “above”) in 1942, refers to the study of any stably inherited phenotypic change that does not rely on alterations in the DNA sequence (Waddington, 2012). Since its first definition, the field of epigenetics underwent a progressive expansion, and it is nowadays widely accepted that epigenetic regulation is of paramount importance in the maintenance of genetic and cellular homeostasis. The ultimate function of epigenetic modifications is to control chromatin accessibility to the transcriptional machinery, thereby regulating the rate of expression of each genetic locus. Control over chromatin accessibility is exerted through a complex network of covalent/noncovalent DNA and histone modifications carried out by a complex protein machinery that involves several players, which can be classically divided into two main categories: *modifiers*, further divided into *writers* and *erasers*, responsible of positioning and removing the covalent chemical modifications, respectively, and *readers*, downstream regulators that recognize and bind specific modifications. Included in the second category, ATP-dependent chromatin *remodelers* are protein complexes responsible for the changes in chromatin accessibility state through noncovalent modifications impacting DNA-histone interactions (e.g., nucleosome sliding or ejection) (Gillette and Hill, 2015). The reversibility of epigenetic modifications ensures a high degree of flexibility, making the epigenome (the complete ensemble of chemical modifications of DNA/histones) an extremely plastic tool capable of spatiotemporally orchestrating gene expression and through which the cell can rapidly and efficiently adapt to environmental changes or respond to specific stimuli.

In the past 20 years, a new player has emerged as one of the fundamental regulators of this complex scenario: non-coding RNA (ncRNA), which can be divided into short non-coding RNA (sncRNA, <200 nt) and long non-coding RNA (lncRNA, >200 nt). The idea that RNA could act as a structural component of chromatin was proposed already in 1975, when Paul and Duerksen noted how the amount of RNA associated with heterochromatin was twice as much as the one associated with euchromatin (Paul and Duerksen, 1975). However, it was only in the past 2 decades, with the development of powerful techniques that allow for deep sequencing of transcriptomes and spatial mapping of RNA and chromatin interactions, that it has been possible to note how almost all the genome is extensively transcribed (Carninci et al., 2005; Djebali et al., 2012) and how some of these transcriptional products have acquired critical regulatory functions.

Indeed, being capable of binding DNA, proteins, and other RNA molecules in a targeted manner, ncRNAs can mediate the interaction of the epigenetic machinery with DNA by sequestering chromatin modifiers by guiding them to the correct genomic location or by serving as molecular scaffolds to coordinate the binding of different interactors (Guttman and Rinn, 2012; Marchese and Huarte, 2014). ncRNAs can bind and regulate the activity of both writers and erasers: for instance, approximately 20% of lncRNAs were shown to interact with the polycomb repressive complex 2 (PRC2), responsible for catalyzing the addition of the repressive mark H3K27me3 (Khalil et al., 2009). Furthermore, RNA can interact with DNA through simple Watson and Crick base pairing, thus forming a heteroduplex, or by inserting into the major groove of the DNA duplex, therefore forming an RNA-DNA triplex structure (Li et al., 2016) that can be recognized by some proteins (Toscano-Garibay and Aquino-Jarquin, 2014) or selectively targeted by small molecules (Arya, 2011). It has been speculated that such structures could have evolved to directly recruit epigenetic factors without needing further protein interactors (Marchese et al., 2017). Depending on the nature of the involved epigenetic regulator, the RNA-binding complex can either promote or repress the expression of the targeted locus (Gendrel and Heard, 2014; Meller et al., 2015).

## 2. RNA-mediated epigenome regulation

### 2.1 Long non-coding RNA-mediated regulation of gene expression

Among the most important mechanisms regulating cell differentiation and embryonic development, the antagonistic action of PRC2 and TrxG/MLL complexes, belonging respectively to the Polycomb (PcG) and Trithorax (TrxG) group proteins, orchestrates the expression of a large number of developmentally regulated genes through controlling the balance between the repressive mark H3K27me3 and the permissive marks H3K4me1/2/3 (Orlando, 2003; Schuettengruber et al., 2017).

**FIGURE 1:**
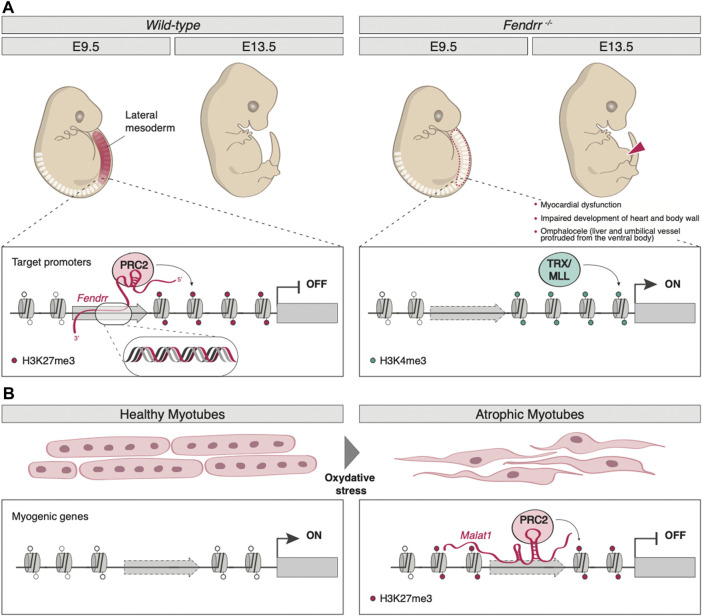
Examples of lncRNA-mediated epigenetic regulation of gene expression. **(A)** By forming an RNA-DNA triplex structure on target promoters (*Foxf1*, *Pitx2,* and *Irx3*), lncRNA *Fendrr* acts as a scaffold that promotes the binding of PRC2-inducing H3K27me3 deposition (left panel). *Fendrr* loss of function results in decreased PRC2 occupancy and increased TRX/MLL-mediated deposition of H3K4me3. The resulting altered epigenetic profile is responsible for severe impairment in heart and body wall development and, ultimately, in embryonic lethality around E13.75 (right panel). **(B)** In muscle, upon oxidative stress, *MALAT1* promotes PRC2 assembly and guides it on the late myogenic genes, inducing their silencing by H3K27me3 deposition.

The lncRNA *Fendrr* (Fetal-lethal non-coding developmental regulatory RNA) was identified as a nuclear-localized transcript, divergently produced from the *Foxf1* gene, whose expression is crucial for the caudal lateral plate mesoderm (LPM) development (Grote et al., 2013). Being capable of binding both PRC2 and TrxG/MLL *in vivo*, as well as the dsDNA in the promoters of the LPM control genes *Foxf1* (in *cis*) and *Pitx2* (in *trans*) *in vitro*, *Fendrr* plays a fundamental role in controlling H3K27me3/H3K4me3 levels at these genomic sites, therefore regulating the expression of its target genes. Indeed, by forming an RNA-DNA triplex structure, *Fendrr* can function as a scaffold that promotes the binding of PRC2, thus antagonizing that of TrxG/MLL, inducing H3K27me3 deposition and repressing the target gene (Figure 1A). In mice, *Fendrr* loss of function results in decreased SUZ12 and EZH2 (two core components of PRC2) occupancy and increased H3K4me3 levels on the promoter of *Foxf1*, *Pitx2*, and *Irx3*, important genes for the control of lateral plate mesoderm (LPM) development; the resulting altered epigenetic profile is responsible for severe impairments in lateral mesoderm differentiation, in heart and body wall development and, ultimately, in embryonic lethality around E13.75 (Grote et al., 2013) (Figure 1A).

In humans, *Fendrr* was found to be significantly downregulated in a wide variety of cancers (Zheng et al., 2021), and its overexpression can reduce proliferative rate, cell migration, and chemoresistance of many tumors (G. Zhang et al., 2018a; Li et al., 2018; Xu and Han, 2019). Therefore, *Fendrr* has been proposed as a candidate diagnostic/prognostic marker, as well as a promising therapeutical target (Zheng et al., 2021). Moreover, *Fendrr* deletion was recently associated with alveolar capillary dysplasia (Kozłowska et al., 2020).

With a mechanism similar to that of *Fendrr*, the maternally expressed gene 3 (*MEG3*) regulates the activity of TGF-β pathway genes by binding distal GA-rich elements through the formation of RNA-DNA triplexes (Mondal et al., 2015). Since *MEG3* was shown to interact with both the PRC2 component EZH2 and the PRC2 recruiter JARID2, it has been proposed that this lncRNA could either directly regulate PRC2 binding and assembly on chromatin or mediate the initial JARID2 recruitment, thus increasing PRC2 recruitment and H3K27 methylation levels (Kaneko et al., 2014). *MEG3* expression has been found to be downregulated in several human tumors (Sun et al., 2016; Gong and Huang, 2017; Dong et al., 2018; Zuo et al., 2020), probably due to the hypermethylation of two regulatory sequences in its promoter (Zhao et al., 2005). Indeed, there is evidence that *MEG3* overexpression could have an anti-tumoral effect, either by suppressing glycolysis through c-Myc degradation (Zuo et al., 2020), by inhibiting Wnt/β-catenin signaling pathway (Gong and Huang, 2017; Zheng et al., 2018) or by enhancing p53 transcriptional activity on its targets (Sun et al., 2016).

*KHPS1* is an antisense lncRNA, transcribed from the bidirectional promoter of the sphingosine kinase 1 (SPHK1), that acts *via* an RNA-DNA triplex to recruit the histone acetyltransferase (HAT) p300/CBP on a poised enhancer, whose transcription promotes SPHK1 expression. Interestingly, the replacement of the triplex-forming region (TFR) of *KHPS1* with that of *MEG3* is enough to guide *KHPS1* on the *TGFBR1*, a gene normally targeted by *MEG3* (Postepska-Igielska et al., 2015; Blank-Giwojna et al., 2019)*.* This observation emphasizes the importance of triplex structures in guiding lncRNAs to specific, distant regions in the genome.

The heart-enriched lncRNA *Chaer* (cardiac hypertrophy-associated epigenetic regulator) binds EZH2 with a 524 nt secondary structure similar to that of *Fendrr* (Wang et al., 2016); however, differently from *Fendrr*, it prevents PRC2 binding to chromatin, thereby decreasing the levels of H3K27me3 at the target genes. *Chaer*-PRC2 interaction is transiently promoted by the mammalian target of rapamycin (mTOR) pathway upon stress or hormonal stimuli and ultimately results in the induction of the genes responsible for heart hypertrophy (Wang et al., 2016). Moreover, a recent work showed that *Chaer* is significantly downregulated in cardiomyocytes of a murine acute myocardial infarction model and that its overexpression, both *in vitro* and *in vivo*, reduces cardiomyocyte apoptosis and heart function impairment through a mechanism dependent on AMP-activated protein kinase (AMPK) phosphorylation (He et al., 2021). Several other lncRNAs have been associated with cardiac mesoderm development, including Braveheart (*Bvht*) and cardiac mesoderm enhancer-associated non-coding RNA (*CARMEN*). The first one interacts with SUZ12 to regulate the expression of several cardiac precursor cells (CPCs) genes, including the master gene *MESP1*, potentially by displacing PRC2 from pro-differentiative genes or by recruiting it to repressors of cardiac differentiation program (Klattenhoff et al., 2013). The second one is a SUZ12/EZH2-interacting critical regulator of CPCs cardiac specification and identity maintenance of mature cardiomyocytes; it is significantly dysregulated in patients suffering from pathological heart remodeling (Ounzain et al., 2015).

Timely and coordinated regulation of gene expression is often achieved through bivalent promoters, kept in a poised state by the deposition of both H3K4me3 and H3K27me3 marks (Bernstein et al., 2006; Voigt et al., 2013). By controlling these bivalent histone marks on the promoter of the pro-apoptotic gene *Bim*, the lncRNA *Morrbid* (myeloid RNA regulator of *Bim*-induced death) is of crucial importance in regulating the lifespan of myeloid cells (Paschos et al., 2012; Kotzin et al., 2016). Specifically, by acting in *cis* through a DNA loop involving its own locus and that of *Bim*, *Morrbid* binds EZH2 and regulates PRC2 occupancy on the *Bim* promoter, thus keeping the pro-apoptotic gene in a poised state (Kotzin et al., 2016). Further evidence of the importance of this lncRNA in myeloid cells homeostasis comes from recent observations linking *Morrbid* dysregulation to the pathogenesis of acute myeloid leukemia (AML) and juvenile myelomonocytic leukemia (JMML) (Cai Aguilera et al., 2020; Cai Zhang et al., 2020).

Further strengthening the association between lncRNAs, PRC2 regulation, and cell identity, pregnancy-induced non-coding RNA (*PINC*) interacts with RbAp46, SUZ12, and EZH2 through evolutionarily conserved loops in its 5’ region to regulate milk proteins production in alveolar cells of the mouse mammary gland; indeed, the decrease in *PINC* expression during the transition from late pregnancy to lactation, in which alveolar cells undergo terminal differentiation upon lactogenic hormone stimulation, suggests that this RNA could act as a break preventing milk production and secretion until parturition (Shore et al., 2012). Moreover, luminal and alveolar progenitor cells surviving mammary gland involution preserve high levels of *PINC*, suggesting that *PINC*/PRC2 joint action could secure the epigenetic maintenance of a progenitor pool with a high differentiation potential which can be rapidly induced to its fate in future pregnancies (Shore et al., 2012).

Some lncRNAs are tightly linked to cell cycle control, such as *GIHCG* (gradually increased during hepatocarcinogenesis) and *PINT* (p53-induced non-coding transcript). *GIHCG* is aberrantly upregulated in hepatocellular carcinoma (HCC), where it recruits both PRC2 and DNA methyltransferase 1 (DNMT1) on the promoter of *miR-200b/a/429*, a microRNA (miRNA) often epigenetically dysregulated in cancer (Wiklund et al., 2011), causing an increase in H3K27me3 and DNA methylation (DNAme) and therefore silencing the locus (Suijun et al., 2016). Aberrant *GIHCG*-mediated miRNAs expression has also been shown to regulate the development and progression of different types of cancer (Fan et al., 2019; Zhao et al., 2020). *PINT* is a lncRNA directly regulated by p53 that, in mice, acts as a positive regulator of cell proliferation (Marín-Béjar et al., 2013). On the other hand, the human ortholog seems to exert the opposite effect, as it has been found significantly downregulated in multiple tumors, interacting with PCR2 to repress invasion-related genes. Indeed, human *PINT* inhibits tumor metastasis when injected in a mouse model of liver cancer (Marín-Béjar et al., 2017). Typically involved in alternative splicing regulation within nuclear speckles (Tripathi et al., 2010), the metastasis-associated lung adenocarcinoma transcript 1 (*MALAT1*) regulates myogenesis through the recruitment of Suv3-9, a histone methyltransferase that catalyzes H3K9me3 deposition, on MyoD promoter (Chen et al., 2017). Upon oxidative stress, *MALAT1* promotes PRC2 assembly and guides it on the late myogenic factors myogenin and myosin heavy chain 8, inducing their silencing (El Said et al., 2021) (Figure 1B). Furthermore, *MALAT1*-PRC2 interaction is also dysregulated in cancer cells, as this lncRNA is typically overexpressed in tumors (Wang et al., 2015; Kim et al., 2017).

So far, we have mainly focused on the significant amount of evidence that associates lncRNAs to PRC2 recruitment/displacement on/from chromatin; nevertheless, these molecules can also influence the activity of the PRC2 antagonists, TrxG/MLL proteins. Specifically, *HOTTIP* and *NeST* are two lncRNAs that have been shown to interact with WDR5, a core component of the H3K4 methyltransferase complexes MLL1-4 and SET1A/1B (Wang et al., 2011; Gomez et al., 2013). *HOTTIP* (*HOXA* transcript at the distal tip), transcribed from the 5′ region of HOXA gene cluster, recruits WDR5/MLL and acts in *cis*, through chromosomal looping, to coordinate the activation of HOXA 5′ genes (Wang et al., 2011). Furthermore, *HOTTIP* aberrant activation alters HOXA cluster 3D structure and induces hematopoietic stem cells to a forced self-renewal, thus promoting AML-like diseases development (Luo et al., 2019). *NeST* (nettoie *Salmonella* pas Theiler’s) is a *trans*-acting RNA expressed in T cells that recruits WDR5 on the interferon-γ locus and therefore regulates the inflammatory response upon microbial infection (Gomez et al., 2013).

Interestingly, some lncRNAs are only expressed under pathological conditions: that is the case of *DBE-T* (D4Z4-binding element transcript), specifically expressed in patients affected by facioscapulohumeral dystrophy (FSHD) and transcribed from the subtelomeric region 4q35, a region found mutated in 95% of FSHD cases; *DBE-T* recruits the TrxG protein Ash1L to de-repress 4q35 genes, that in adult cells are normally silenced by H3K27me3 marks, and that in FSHD are aberrantly re-expressed due to a TrxG/PcG epigenetic switch consisting of a loss in H3K27me3 paralleled by an AshL1-mediated H3K36me2 deposition (Cabianca et al., 2012). Moreover, Ash1L induces the expression of *DBE-T*, leading to a positive feedback loop that maintains 4q35 genes in a constitutively de-repressed state (Cabianca et al., 2012).

In the last decade, the finding that enhancers can be actively transcribed led to the identification of a heterogenous class of regulatory non-coding transcripts, termed enhancer RNAs (eRNAs), whose importance in gene regulation has often been debated. eRNAs are short (median 346 nt), unstable, and unspliced nuclear-retained transcripts, bidirectionally transcribed by RNAPII from regions marked with the permissive marks H3K4me1 and H3K27ac (Andersson et al., 2014). The identification of enhancers relies on common features, including unique epigenomic signatures, ncRNA transcription, and TF binding sites (Natoli and Andrau, 2012; Ong and Corces, 2012; Calo and Wysocka, 2013; Furlong and Levine, 2018). However, the bioinformatics-based genome-wide predictions do not prove the functional aspects of *in silico*-identified enhancers. Systematic gain- or loss-of-function CRISPR-based technology is the gold standard approach to provide the experimental proof of the actual functional potential of these non-coding elements. The genome-wide production of eRNAs could lead to considering these elements as the mere product of transcriptional noise. However, several studies strengthened the hypothesis that eRNAs transcription is a conserved phenomenon with a well-defined function. Indeed, it is nowadays evident that many of the identified eRNAs can promote tissue- and cell-specific gene expression (Kim et al., 2015; Han and Li, 2022) through several mechanisms such as strengthening enhancer-promoter looping (Lai et al., 2013; Li et al., 2013), regulating the binding of RNAPII to promoters (Sigova et al., 2015; Rahnamoun et al., 2018) or modulating transcriptional elongation (Schaukowitch et al., 2014; Zhao et al., 2016).

Finally, as previously shown for *CARMEN* (Ounzain et al., 2015), some eRNAs can regulate chromatin accessibility through interaction with chromatin modifiers enzymes such as EZH2, SUZ12, and p300/CBP (Bose et al., 2017). The interaction with CBP, in particular, has been shown to promote the actuation of the myogenic differentiation program and seems to be mediated by the nuclear Argonaute1 (Ago1), a key component of RNA-interference (RNAi) pathway (Fallatah et al., 2021). Notably, Ago1 depletion leads to the disruption of global chromatin architecture, and its enrichment at enhancer sequences seems to be dependent on rRNAs expression (Shuaib et al., 2019). Such observations collectively highlight the importance of these little-known regulatory RNAs in coordinating gene expression and shaping chromatin three-dimensional structure.

### 2.2 Epigenetic control of X-chromosome inactivation: *Xist*

Identified in 1991, the X-inactive-specific transcript (*Xist*) was one of the first lncRNAs to be discovered. It plays a pivotal role in the X-chromosome inactivation (XCI) process, evolved in therian mammals as a mechanism for compensating the dosage of X-related genetic products in XX individuals (Brown et al., 1991). XCI consists of a series of modifications of the inactive X-chromosome (Xi), which undergoes epigenetic modifications, structural reorganization, and spatial repositioning, ultimately becoming transcriptionally silent. XCI is initiated by the monoallelic expression of *Xist*, whose locus is situated in the X inactivation center (Xic) on the X-chromosome. The “choice” between the two *Xist* alleles appears to be random, and the process can be initiated only when Xic is present on at least two chromosomes. Once induced, *Xist* completely coats the X-chromosome from which it is expressed, causing its progressive inactivation (Augui et al., 2011). Indeed, *Xist cis* action is so powerful that it can drive the inactivation of autosomes on which the lncRNA is ectopically expressed (Lee and Jaenisch, 1997).

*Xist* interacts with several proteins to orchestrate XCI. Although the precise order of events is not fully understood, one necessary *Xist* interactor is SHARP, which has been shown to recruit both the transcriptional repressor SMRT and the histone deacetylase 3 (HDAC3) to exclude RNA polymerase II (RNAPII) from the *Xist*-coated X-chromosome (McHugh et al., 2015). Interestingly, the maintenance of the transcriptional repression on Xi is thought to be achieved by PRC1/PRC2 sequential action. Specifically, one of the proposed mechanisms is that *Xist* can first recruit a non-canonical PRC1 *via* the interactor hnRNPK, and then PRC1 catalyzes the monoubiquitination of the histone H2A on lysine 119 (H2AK119ub), a modification that can be recognized by JARID2, that in turn recruits PRC2. The subsequent H3K27me3 deposition serves as a signal for the binding of canonical PRC1, which reinforces the transcriptionally silent state through more H2AK119ub deposition (Galupa and Heard, 2018).

### 2.3 The epigenetic control of rRNA and telomeres: pRNA and *TERRA*

**FIGURE 2:**
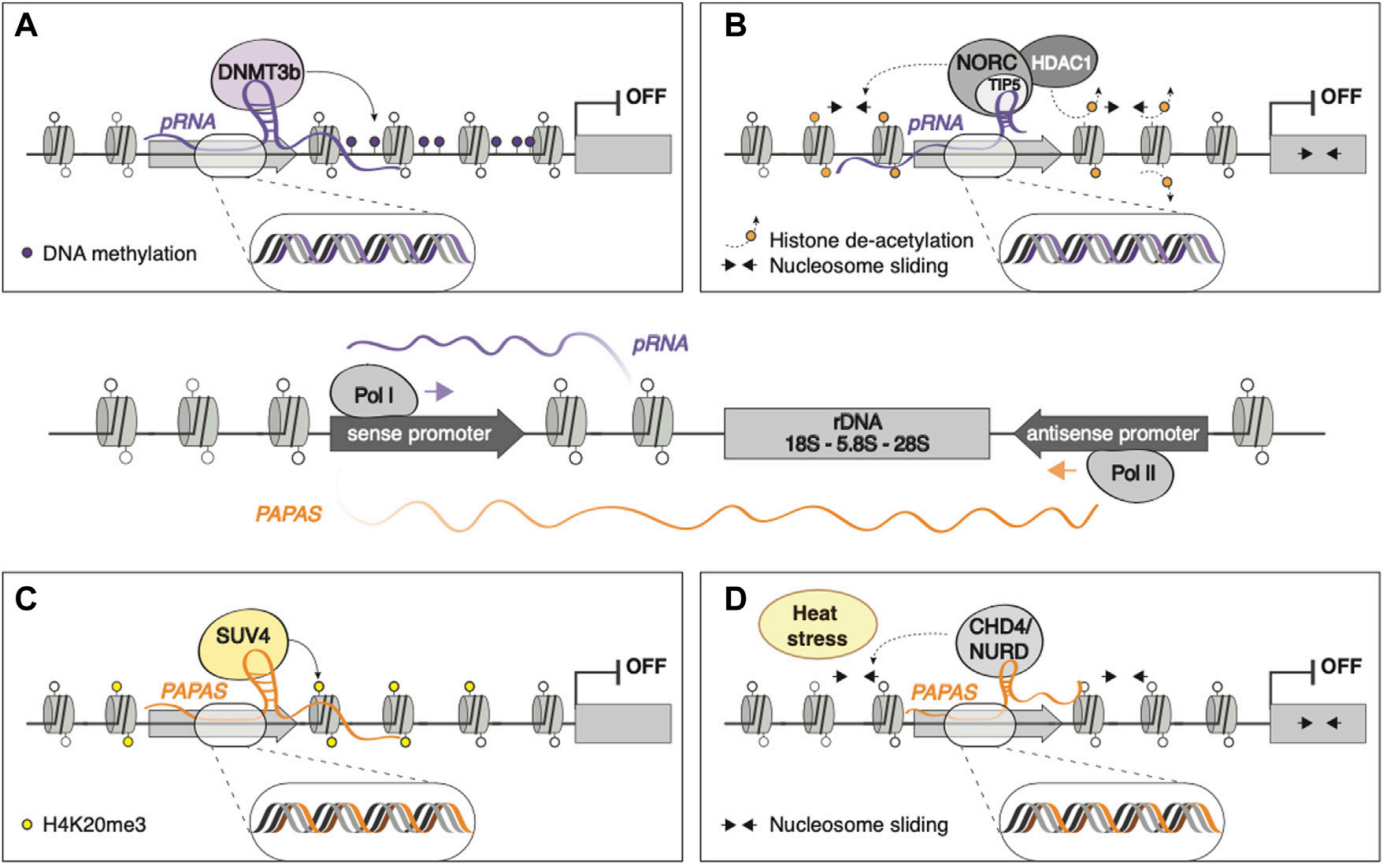
The epigenetic control of rRNA: pRNA and *PAPAS* RNAPI binding to rDNA promoter gives rise to the transcription of pRNA, whose sequences match that of rDNA promoter. In contrast, the antisense transcription of rDNA, mediated by RNAPII, gives rise to *PAPAS*, spanning from rDNA gene body to intergenic, promoter, and enhancer regions (Figure, central panel). pRNA can mediate the silencing of rDNA loci by **(A)** directly recruiting DNMT3b, leading to *de novo* methylation and heterochromatinization; **(B)** binding NoRC and HDAC, promoting nucleosome sliding and deacetylation, respectively. **(C)** *PAPAS* leads to rDNA silencing by recruiting SUV4 on rDNA promoter. **(D)** *PAPAS* can associate with the chromatin remodeling complex CHD4/NuRD and guide it on the rDNA locus to promote nucleosome shifting and transcriptional repression of rDNA in a heat stress-dependent manner.

Eukaryotic ribosomal RNA genes (rDNA) are present in tandem repeats throughout the genome, transcribed from RNA polymerase I (RNAPI) promoters that in mice are located ∼2 kb upstream rRNA transcription start site (Mayer et al., 2006). RNAPI binding to rDNA promoter often leads to the transcription of an intergenic RNA that is rapidly processed into 150–250 nt fragments, termed promoter-associated RNA (pRNA), whose sequences match that of rDNA promoter (Figure 2, central panel). These fragments are protected from degradation by TIP5 (TTF-I-interacting protein 5) binding (Mayer et al., 2008), the major subunit of the nucleolar remodeling complex (NoRC), an ATP-dependent chromatin remodeling complex that has been shown to repress rRNA transcription through H4 tail-dependent nucleosome sliding and HDAC1 recruitment (Strohner et al., 2001; Zhou et al., 2002) (Figure 2B). pRNA can also directly recruit DNA methyltransferase DNMT3b on rDNA promoter by forming an RNA-DNA triplex at the TTF-I transcription factor binding site, leading to *de novo* methylation and heterochromatinization of rDNA genes (Schmitz et al., 2010) (Figure 2A).

The antisense transcription of rDNA can give rise to RNAPII-transcribed, >10 kb long regulatory lncRNAs, termed *PAPAS* (promoter and pre-rRNA antisense), that span from rDNA gene body to intergenic, promoter, and enhancer regions (Figure 2, central panel). *PAPAS* are upregulated in growth-arrested cells and downregulated in cancerous cells, guides the histone methyltransferase Suv4-20h2 to rDNA promoter, leading to chromatin compaction and transcriptional silencing of rDNA genes through H4K20me3 deposition (Bierhoff et al., 2014) (Figure 2C). Furthermore, *PAPAS* can associate with the chromatin remodeling complex CHD4/NuRD and guide it on the rDNA locus to promote nucleosome shifting and transcriptional repression of rDNA in a heat stress-dependent manner (Zhao et al., 2018) (Figure 2D).

pRNA and *PAPAS* are examples of cis-acting TSS-associated ncRNAs (Guil and Esteller, 2012), divergently transcribed RNAs from promoter regions identified across the mammalian genome by global nuclear run-on sequencing (GRO-seq) (Core et al., 2008; Seila et al., 2008). The general rules about the regulation of opposite transcription and its influence on neighboring coding genes, however, are still unclear. The observed correlation between pRNA and mRNA expression level suggests a shared mechanism of transcriptional regulation. However, it cannot be excluded that this tendency is a direct consequence of their proximity and subsequent shared local chromatin conformation and regulatory features.

Given their repetitive nature, rDNA clusters can be responsible for abnormal homologous recombination: indeed, 50% of solid tumors show rDNA-driven genomic alterations (Stults et al., 2009). Hence, the coordinated activity of 6 and *PAPAS*, and therefore a correct rDNA-containing chromatin regulation, is crucial for cellular homeostasis.

Telomere length sensing and regulation is critical for cell cycle dynamics: too short telomeres are thought to be an indicator of the onset of cellular senescence, whereas aberrant re-elongation occurs in almost all forms of cancer (Shay and Wright, 2019). Telomere re-elongation can be achieved through two main mechanisms: telomerase re-expression (85%–90% of human cancers) or alternative lengthening of telomeres (ALT), a mechanism that exploits homology-directed repair (HDR) and that is present in 10%–15% of tumors (Shay and Wright, 2019).

Telomeric DNA is usually associated with a particular protein complex named shelterin, which protects the integrity of chromosome ends by preventing the accidental activation of DNA damage pathways (de Lange, 2018). Moreover, telomeres are marked with the heterochromatin histone modifications H3K9me3 and H4K20me3 (García-Cao et al., 2004; Benetti et al., 2007). Given its heterochromatic nature, telomeric DNA was traditionally thought to be transcriptionally silent. Thus, it was not without a certain surprise to discover that these regions are actively transcribed by RNAPII into a telomeric UUAGGG repeat-containing RNA (*TERRA*), ranging from 100 bp to 9 kb, that originates from subtelomeric CpG islands that serve as promoters (Azzalin et al., 2007; Nergadze et al., 2009). Since *TERRA* depletion results in the loss of heterochromatic marks and structural abnormalities of metaphasic chromosome ends, this RNA appears to have the important function of heterochromatin maintenance at telomeres, thus ensuring their structural integrity (Deng et al., 2009). Furthermore, similarly to what has been observed for pRNA, *TERRA* interacts with TIP5 and is therefore likely to act as a coordinator of NoRC-dependent heterochromatinization of telomeres. Indeed, TIP5 directly interacts with Suv3-9, Suv4-20h2, and sirtuin 6 (Sirt6), a histone deacetylase, and its overexpression or knockdown lead to an increase or a diminution, respectively, of H3K9me3, H4K20me3, and hypoacetylated H4 (Postepska-Igielska et al., 2013). Finally, *TERRA* is required for PRC2-dependent H3K27me3 deposition whose loss results in impaired deposition of H3K9me3, H4K20me3, and heterochromatin protein 1 (HP1) (Montero et al., 2018).

*TERRA* can directly bind one of the two telomeric DNA strands, forming a particular secondary structure, termed R-loop, composed of the RNA-DNA hybrid and the unbound DNA strand. Interestingly, it has been proposed that the accumulation of *TERRA*-induced RNA-DNA hybrids may serve as a trigger for homology-directed repair (HDR), thus promoting alternative lengthening of telomeres (ALT) (Arora and Azzalin, 2015). In fact, when the length of a telomere becomes critically short, an impairment in *TERRA* degradation causes R-loops to locally accumulate and induce the re-elongation of the short telomere through HDR, thus preventing premature replicative senescence (Graf et al., 2017). In human cells, *TERRA* expression is strictly regulated by cell cycle, with a peak phase corresponding to early G1 (Porro et al., 2010), and is altered in cancer cells that exploit ALT (Flynn et al., 2015), thus suggesting a potential therapeutical target for this particular class of tumors.

### 2.4 *Kcnq1ot1* and *Airn*: RNA-mediated chromatin imprinting

Genetic imprinting is an intergenerational epigenetic mechanism that ensures the parent-of-origin-dependent expression of one of two alleles. Usually, imprinted genes are organized in clusters, each individually regulated by an imprinting enter, that share common patterns of differentially methylated regions (DMRs) or histone modifications. These modifications are established in the mature germ cells of an individual and last for one generation, being eventually erased in the germline precursor of their offspring (Monk et al., 2019). Imprinted loci regulation is exerted by *cis*- or *trans*-acting factors such as transcription factors or lncRNAs such as *Kcnq1ot1* and *Airn*.

*Kcnq1ot1* is a 91 kb RNAPII-transcribed RNA oriented in antisense to the potassium voltage-gated channel subfamily 1 (*KCNQ1*) gene, located at human chromosome 11p15.5, that is exclusively expressed on the paternal chromosome, as its control region is methylated on the maternal chromosome (Smilinich et al., 1999). *Kcnq1ot1* is expressed in all body tissues, where it promotes the silencing of a cluster of ubiquitously imprinted genes in the KCNQ1 domain, possibly through interaction with DNMT1, RNAi, or transcriptional interference (Mancini-DiNardo et al., 2006; Mohammad et al., 2010). However, *Kcnq1ot1* also mediates the imprinting of a distal set of genes that are selectively silenced in placental tissue (Pandey et al., 2008). This lineage-specific bidirectional imprinting is thought to be achieved by the selective interaction of *Kcnq1ot1* with both H3K9 methyltransferase G9a and EZH2/SUZ12, observed in placenta but not in fetal liver (Pandey et al., 2008). Impairments in 11p15.5 imprinting have been associated with two growth disorders, Silver-Russel syndrome and Beckwith-Wiedemann syndrome (Smilinich et al., 1999; Chiesa et al., 2012), and aberrations in *Kcnq1ot1* expression were identified in a plethora of human cancers, where it interferes with several cellular processes mainly by acting as a competing endogenous RNA (ceRNA), regulating various miRNAs availability through sponging mechanisms (Cagle et al., 2021).

The mouse-specific lncRNA *Airn*, located in the second intron of the insulin-like growth factor 2 receptor (*Igf2r*) gene in an antisense orientation, is imprinted and only expressed from the paternal chromosome, where it silences in *cis* its target genes *Igf2r* and the solute carrier family 22 members *Slc22a2* and *Slc22a3*. Specifically, *Igf2r* is silenced in embryonic, extraembryonic, and adult tissues, whereas the other two genes are only silenced in some extraembryonic tissues (Sleutels et al., 2002; Hudson et al., 2011). Similarly to *Kcnq1ot1*, the two mechanisms through which *Airn* operates to repress gene expression seem to differ depending on the location of its targets: if the distal target *Slc22a3* silencing is achieved through G9a recruitment and H3K9me deposition (Nagano et al., 2008), *Igf2r* is repressed *via* transcriptional interference (TI), which precludes RNAPII recruitment on its promoter (Latos et al., 2012).

### 2.5 Non-coding natural antisense transcripts

The previously shown lncRNAs *Kcnq1ot1* and *Airn* share a common feature: they overlap with their target transcript. These RNAs are three examples of natural antisense transcripts (NATs), a highly abundant and heterogenous class of RNAs that was possible to characterize in the last 20 years through the development of powerful techniques such as cap analysis gene expression (CAGE) and single strand RNA sequencing (ssRNA-Seq) (Katayama et al., 2005; Balbin et al., 2015). NATs are defined as coding or non-coding transcripts whose sequence is complementary or overlaps with that of a protein-coding or non-coding transcript (Balbin et al., 2015). NATs can be transcribed from cryptic promoters, usually located in introns, and can function in *cis* or *trans*. Since the vast majority of the genome is pervasively transcribed and about 98.8% of the mammalian genome is composed of non-coding sequences, most NATs do not code for any protein and are therefore termed non-coding NATs (ncNATs).

One of the hypothesized mechanisms through which ncNATs can regulate gene expression is TI. If widespread, a negative correlation between sense-antisense pairs expression should be expected since TI is thought to be a downregulatory mechanism. Nevertheless, the majority of significant correlations between the expression of the antisense transcripts and that of their corresponding sense transcripts seem to be positively linked (Oeder et al., 2007; Krappinger et al., 2021). Indeed, to date only few ncNATs have been proven to act through TI.

Other ncNATs control gene expression through chromatin modifiers enzymes recruitment: particularly relevant examples include the oncogenic lncRNAs *HOTAIR*, *ANRIL*, *CCAT2,* and *TUG1*, all of which interact with PRC2 subunits (Rinn et al., 2007; Khalil et al., 2009; Yap et al., 2010; Deng et al., 2017), and *GPC3-AS1*, which interacts with p300/CBP (Zhu et al., 2016). *HOTAIR*, transcribed from the HOXC locus, associates with both PRC2 and lysine-specific demethylase 1 (LSD1), a subunit of the histone demethylase/deacetylase CoREST repressor complex, to guide them on the HOXD locus, thus exerting a dual inhibitory control by simultaneously adding repressive marks and removing permissive ones (Rinn et al., 2007; Tsai et al., 2010). The other three PRC2-interacting lncRNAs are epigenetic silencers of various cyclin-dependent protein kinase inhibitors such as p15, p16, p21, and p57, thus being deeply involved in the regulation of cell cycle progression, cellular senescence, and apoptosis (Yap et al., 2010; Zhang et al., 2016; Deng et al., 2017). On the other hand, *GPC3-AS1* functions as an activator, promoting an increase in euchromatic marks on *GPC3* gene body, thereby increasing its transcription (Zhu et al., 2016). All of the abovementioned ncNATs can also function as ceRNAs, balancing (or unbalancing, when dysregulated) the levels of a wide variety of different miRNAs involved in cancer onset and progression (Bhan and Mandal, 2015; Xie et al., 2017; Zhang J.-J. et al., 2018; Zhou et al., 2019). Unsurprisingly, these ncNATs have been found to be upregulated in several tumors (Zhu et al., 2016; Krappinger et al., 2021).

An interesting example of how ncNATs can establish highly complex regulatory networks comes from the works characterizing the mechanism through which phosphatase and tensin homolog pseudogene (*PTENpg1*) regulates *PTEN* expression both at the transcriptional and post-transcriptional levels. *PTENpg1* is transcribed in both senses, giving rise to different ncRNAs: a sense transcript and two antisense RNAs, *PTENpg1 asRNA α* and *PTENpg1 asRNA β*. The sense transcript, *PTENpg1*, acts in the cytoplasm as a sponge for miRNAs that would otherwise target *PTEN* mRNA, thus increasing its stability. However, it lacks a poly(A) tail, and thus it cannot be efficiently exported from the nucleus. On the other hand, the two antisense RNAs are polyadenylated and exert two different functions: the *α* isoform guides PRC2 and DNMT3a on *PTEN* 5’ untranslated region (UTR), located to a different chromosome, to repress its transcription in *trans*; the *β* isoform binds *PTENpg1*, favoring its nuclear export, through the formation of an RNA-RNA duplex whose poly(A) can be recognized by the nuclear transport machinery (Poliseno et al., 2010; Johnsson et al., 2013). Therefore, two ncNATs transcribed from a single pseudogene operate a simultaneous and opposite control over *PTEN* expression, thereby regulating the abundance of this critical tumor-suppressor. *PTENpg1* is not the only pseudogene-derived, *trans*-acting RNA shown to epigenetically regulate target genes. Indeed, Oct4, one of the four Yamanaka pluripotency factors, is negatively regulated by the product of the antisense transcription of one of its six pseudogenes (Hawkins and Morris, 2010; Lister et al., 2021).

ncNATs represent an extraordinarily abundant source of regulatory molecules that deserves to be further explored. Their involvement in a growing number of human diseases makes them promising candidates for both diagnosis procedures and therapeutical approaches (Krappinger et al., 2021). Therefore, it will be crucial to investigate in detail the epigenetic and non-epigenetic mechanisms through which these molecules exert their control over gene expression.

### 2.6 RNA in chromatin architecture organization

The nucleus possesses a tightly regulated three-dimensional (3D) structure established by how chromosomes are folded and bound by sub-nuclear structures. In recent years, the development of powerful high-throughput techniques, such as Hi-C, allowing to map physical interactions between chromosome portions, led to the observation that interphasic chromatin is hierarchically organized into high-order structures such as topologically-associated domains (TADs), lamina-associated domains (LADs) and chromatin loops (Pombo and Dillon, 2015; Rowley and Corces, 2018). LADs are H3K9me2/H3K9me3-enriched, little-transcribed chromatin regions that localize in the nuclear periphery and whose formation is mediated by the interactions between the nuclear lamina and chromatin-binding proteins (Briand and Collas, 2020). TADs, on the other hand, are structures spanning up to 1 Mb that interact more with themselves than with the rest of the genome, whose boundaries are enriched in CCCTC-binding factor (CTCF) and constitutively transcribed housekeeping genes (Dixon et al., 2012). Each TAD can encompass multiple loops, each one established by cohesin-mediated chromatin winding, whose extrusion is blocked by CTCF binding (Mirny and Solovei, 2021). By spatially constraining a portion of the genome, CTCF-mediated chromatin looping can influence gene expression both by favoring preferential interactions between enhancers and promoters within the loop and by shielding these elements from regulatory sequences lying outside the loop. Nevertheless, the fact that CTCF can also be recruited by transcription factors and that active transcription plays a fundamental role in defining TADs boundaries indicates that instead of being two hierarchical levels of gene regulation, 3D chromatin structure and transcription are strongly interdependent on each other (Rowley and Corces, 2018).

The large amount of RNAs associated with chromatin suggests an important function of these molecules in chromosomal structure maintenance. Indeed, already in 1989, it was evident that a global RNA depletion results in profound nuclear str abnormalities (Nickerson et al., 1989). The ability to interact with both DNA and other RNA molecules, as well as with proteins, makes RNA a versatile scaffold, efficiently serving as a bridge to coordinate the assembly of various nuclear compartments. However, performing a genome-wide analysis of RNA distribution in nuclear compartments has been extremely challenging. The difficulty lies in the fact that neither traditional proximity ligation- nor fluorescence microscopy-based methods allow for a high-throughput, simultaneous mapping of RNA-DNA, RNA-RNA, and DNA-DNA interactions. This problem was recently overcome by the development of RD-SPRITE, a promising technique that provided an important experimental proof of the mechanisms through which RNA shapes chromatin architecture (Quinodoz et al., 2021).

The data emerging from RD-SPRITE experiments reveal an entangled scenario, in which some transcripts, such as *MALAT1,* are diffusely present on chromatin, whereas others form spatially concentrated “hubs” harboring functionally related ncRNAs that often co-localize with the genomic loci from which these RNAs are transcribed. This last category includes transcripts involved in heterochromatin formation, gene expression regulation, and RNA processing, such as *Kcnq1ot*, that localizes in a TAD encompassing all the imprinted genes and excluding the non-imprinted ones, *Airn*, *Xist,* and the small nuclear RNA (snRNA) *U7* (Quinodoz et al., 2021). Notably, blocking transcription interferes with the assembly of these nuclear hubs suggesting that the nascent RNA could play a pivotal role in establishing these structures. Indeed, the authors hypothesized a mechanism in which the accumulation of nascent transcripts in proximity to their transcription sites serves as a “seed”, leading to the progressive recruitment of diffusible ncRNAs and proteins to create a spatially defined compartment (Quinodoz et al., 2021).

### 2.7 Epigenetic regulation by small non-coding RNAs

In the last couple of decades, small non-coding RNAs (sncRNAs), defined as non-coding transcripts shorter than 200 nt, emerged as critical players in all aspects of gene expression regulation, including epigenetic modulation.

Generated from dsRNA precursors, cleaved by Dicer, and loaded onto Ago proteins, miRNAs are ∼24 nt RNA molecules that commonly bind to the 3′UTR of mRNAs, causing their translational repression or, if a high degree of complementarity between the miRNA seed sequence and the target is achieved, their cleavage and degradation (O’Brien et al., 2018). miRNA can exert their post-transcriptional control over mRNAs of several enzymes involved in all aspects of epigenetic regulation, influencing both DNA methylation and histone modification levels (Chen et al., 2006; Fabbri et al., 2007; Wang et al., 2008; Garzon et al., 2009; Roccaro et al., 2010; Yuan et al., 2011; Takeshima et al., 2020). However, as they only regulate the global level of a given epigenetic modifier, they do not directly exert a regulation aimed at the final target loci. In contrast with miRNAs, piRNAs are generated from ssRNA precursors in a Dicer-independent manner. They are only expressed in the germ line of most animals, where they maintain genomic integrity by silencing transposons, specifically by cleaving their RNAs through the interaction with PIWI proteins (Ozata et al., 2019). Besides the canonical anti-transposon function, some authors proposed that the PIWI-piRNA complex could act as a transcriptional activator in *Drosophila*, favoring the recruitment of chromatin modifiers, promoting the euchromatinization of some specific sub-telomeric heterochromatic regions (Yin and Lin, 2007). Additionally, PIWI depletion in mice results in the loss of DNAme on transposons loci, suggesting a role of PIWI-piRNA complex in repressing mobile elements through an epigenetic pathway parallel to the classical RNAi (Carmell et al., 2007). Despite these encouraging findings, further evidence is needed to elucidate the role of sncRNAs as epigenetic regulators.

### 2.8 Circular RNAs

Circular RNAs (circRNAs) are covalently closed RNA molecules that originate from a particular form of splicing termed backsplicing, in which a downstream donor site is linked to an upstream acceptor site. This is often due to physical constraints imposed by the self-recognition of two complementary inverted repeats (e.g., Alu elements) flanking the two splicing sites (Jeck et al., 2013).

The absence of free 5′/3′-OH ends makes circRNAs extremely stable molecules, with a half-life (median 18.8–23 h) at least 2.5 times longer than linear transcripts (Enuka et al., 2016), so stable that they have provocatively been proposed as molecular memory tools that neurons can exploit to keep track of synaptic events (Rybak-Wolf et al., 2015).

**FIGURE 3:**
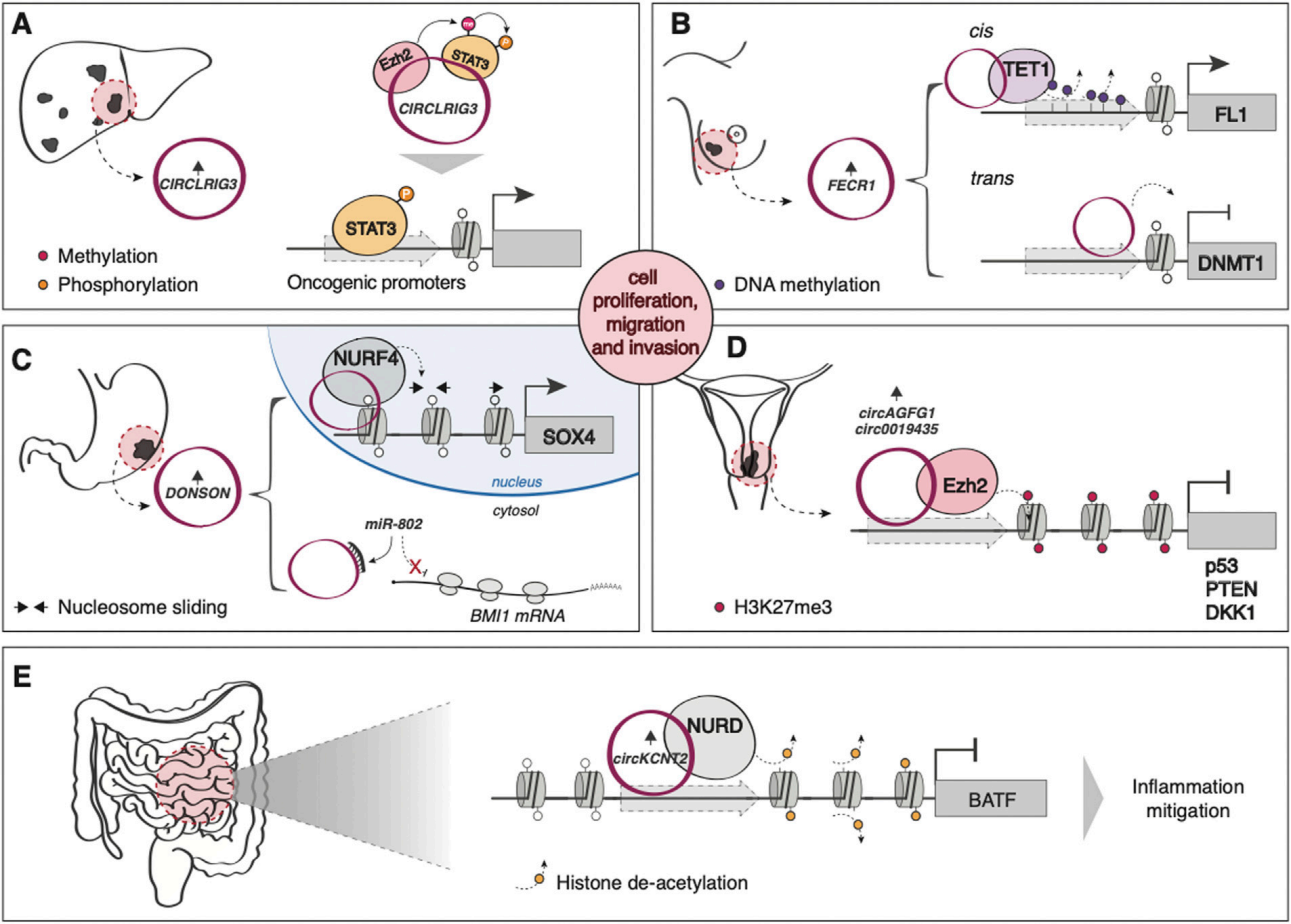
Examples of circRNA-mediated epigenetic regulation of gene expression **(A)** In hepatocellular carcinoma (HCC), the upregulated *circLRIG3* acts as a scaffold to promote the interaction between EZH2 and its substrate, STAT3. Methylated and, subsequently, phosphorylated STAT3 induces the expression of oncogenic gene targets. **(B)** In breast cancer, *FECR1* simultaneously recruits TET1 (in cis) and represses DNMT1 (in trans) to decrease the methylation levels of the oncogene Friend leukemia virus integration 1 (FLI1). **(C)** circRNA *DONSON*, overexpressed in gastric cancer, modulates the levels of BMI1 by sponging miR-802 in the cytoplasm while inducing the nuclear expression of SOX4 by directly recruiting NURF on its promoter. **(D)** circAGFG1 and circ0019435 are both overexpressed in cervical cancer and induce the EZH2-mediated silencing of oncosuppressors p53 and PTEN, and DKK1, respectively. **(E)** *circKcnt2* is overexpressed upon intestinal inflammation and mitigates the inflammatory response through the recruitment of NuRD complex on the promoter of the pro-inflammatory molecule BATF.

While they have traditionally been associated with miRNA sponging functions (Kristensen et al., 2019), recent evidence indicates that circRNAs can interact with different chromatin modifiers and remodelers. For example, many circRNAs have been shown to promote tumorigenesis by modulating EZH2 activity, either by directly recruiting it to suppress gene expression (Zheng et al., 2019; Wang and Li, 2020) (Figure 3D), by regulating the levels of miRNAs targeting the PCR2 subunit (Wang et al., 2019; Yang et al., 2020) or by acting as a scaffold to promote the interaction between EZH2 and its substrate, STAT3 (Sun et al., 2020) (Figure 3A). Another circRNA, *FECR1*, is able to simultaneously recruit TET1 and repress DNMT1 to decrease the methylation levels of the oncogene Friend leukemia virus integration 1 (FLI1), aberrantly overexpressed in various tumors, promoting metastasis in breast cancer (Chen et al., 2018) (Figure 3B). circRNA *DONSON*, overexpressed in gastric cancer (GC), modulates the levels of the PRC1 component BMI1 *via* miR-802 sponging (Y. Liu et al., 2020b) and promotes CG proliferation and invasion by regulating SOX4 expression through directly recruiting the ATP-dependent nucleosome remodeling factor (NURF), a chromatin remodeling complex (Ding et al., 2019) (Figure 3C). *circKcnt2*, on the other hand, is overexpressed upon intestinal inflammation and mitigates the inflammatory response through the recruitment of the nucleosome remodeling deacetylase (NuRD) complex on the promoter of the pro-inflammatory molecule BATF (B. Liu et al., 2020a) (Figure 3E).

## 3 Conclusion and remarks

A significant fraction of the genome-wide transcription of non-protein coding RNAs is involved in nuclear regulatory processes. Particularly, ncRNAs have emerged as pivotal players in the epigenetic regulation of gene expression. Indeed, being physically associated with chromatin, various RNA are crucial components of chromatin in eukaryotes as well as critical constituents of ribonucleoprotein complexes involved in the modulation of local and global chromatin states (Guttman and Rinn, 2012; Cech and Steitz, 2014; Meller et al., 2015).

RNA has the unique feature of being able to bind both protein and nucleic acid. Sequence-based and structure-based molecular interactions are promoted by specific sequence motifs as well as by a variety of secondary and high-order structures, respectively. On the other hand, RNA can bind specifically and directly to other nucleic acids as a triple helix or R-loop. Binding flexibility and target specificity make RNA the most suitable molecule to bring functions exerted, for instance, by chromatin modifiers, to specific genomic targets. Indeed, through being either retained in *cis* at their site of transcription or recruited in *trans* to other loci, RNA is potentially able to reach any genomic region and mediate the modulation of its chromatin architecture, resulting in an altered transcriptional readout. Despite the recently developed technologies that advanced our understanding of RNA-mediated modulation of chromatin state, many aspects of this complex field are still debated. For instance, biochemical and molecular details of the RNA-binding specificity of PRC2 and its regulation by the large number of lncRNA interacting with it are still missing (Brockdorff, 2013; Davidovich and Cech, 2015; Ringrose, 2017). Additionally, more attention is needed before concluding that crosstalk exists between any interacting ncRNA and chromatin modifiers (Blanco and Guttman, 2017), as suggested by a recent finding that PRC2 is dispensable for *HOTAIR*-mediated transcriptional repression (Portoso et al., 2017).

The large amount of RNAs associated with chromatin suggests an essential function of these molecules also in chromosomal structure maintenance. The ability to interact with both DNA and other RNA molecules, as well as with proteins, makes RNA a versatile scaffold, efficiently serving as a bridge to coordinate the assembly of various nuclear compartments. Recently, the development of RD-SPRITE provided important experimental proof of the mechanisms through which RNA shapes chromatin architecture (Quinodoz et al., 2021). The data emerging from RD-SPRITE experiments reveal an entangled scenario in which some transcripts, such as *MALAT1,* are diffusely present on chromatin, whereas some others form spatially concentrated “hubs,” harboring functionally related ncRNAs that often co-localize with the genomic loci from which these RNAs are transcribed. This last category includes transcripts involved in heterochromatin formation, gene expression regulation, and RNA processing, such as *Kcnq1ot*, *Airn*, and *Xist* (Quinodoz et al., 2021).

The first evidence of the importance of RNA in maintaining nuclear structure and activity was provided in 1989 (Nickerson et al., 1989). Nowadays, although many mechanisms are not fully understood, we have a much more detailed view of the countless ways in which RNA mediates the regulation of gene expression, particularly by directly participating in the epigenetic mechanisms.
